# Chemical Composition, Antioxidant Activity, and Multivariate Analysis of Four Moroccan Essential Oils: *Mentha piperita, Mentha pulegium, Thymus serpyllum,* and *Thymus zygis*

**DOI:** 10.1155/tswj/5552496

**Published:** 2024-11-27

**Authors:** Mohamed Amine El Maimouni, Soukaina El Amrani, Mouhcine Fadil, Naoual Menyiy, Rabia Bouslamti, Saoussan Annemer, Sanae Lairini, Abdelhakim El Ouali Lalami

**Affiliations:** ^1^Materials, Processes, Catalysis and Environment Laboratory, Higher School of Technology of Fez, Sidi Mohamed Ben Abdellah University, Imouzzer Road, Fez 30000, Morocco; ^2^Physio-Chemical Laboratory of Organic and Inorganic Materials, Materials Science Center (MSC), Ecole Normale Supérieure, Mohammed V University in Rabat, Rabat, Morocco; ^3^Pharmacology Laboratory, National Agency for Medicinal and Aromatic Plants, 159 Taounate Principale, Taounate 34000, Morocco; ^4^Laboratory of Applied Organic Chemistry, Faculty of Sciences and Techniques, University Sidi Mohammed Ben Abdellah, Fez, Morocco; ^5^Higher Institute of Nursing Professions and Health Techniques of Fez, Regional Direction of Health, El Ghassani Hospital-Dhar El Mehraz, Fez 30000, Morocco

**Keywords:** ABTS^+^, DPPH, food antioxidants, FRAP, H_2_O_2_, *Mentha* species, TAC, *Thymus* species

## Abstract

**Background:** Food chemical antioxidants have demonstrated protective effects against reactive oxygen species and free radicals, but present in excess, harmful consequences might occur on health. Therefore, replacing these synthetic additives with nontoxic natural antioxidants is crucial.

**Objective:** The current study examined aroma profile, antioxidant activity, and multivariate analysis of *Mentha piperita*, *Mentha pulegium*, *Thymus serpyllum*, and *Thymus zygis* essential oils from Morocco.

**Methods:** GC-MS analysis was carried out to determine the chemical composition of the four oils, and their antioxidant activity was evaluated with 2,2-diphenyl-1-picrylhydrazyl (DPPH), cation radical (ABTS^+^), hydrogen peroxide scavenging capacity (H_2_O_2_), ferric reducing antioxidant power (FRAP), and total antioxidant capacity (TAC) methods.

**Results:** Isomintlactone (35.55%), pulegone (74.04%), borneol (37.87%), and borneol (30.99%) were the most abundant compounds of *M. piperita, M. pulegium*, *T. serpyllum*, and *T. zygis* EOs. The antioxidant activity of the four EOs was particularly notable, with an IC_50_ varying between 3.51 ± 0.22 mg/mL and 0.49 ± 0.08 mg/mL by the DPPH method, 1.02 ± 0.21 mg/mL and 0.4 ± 0.7 mg/mL by the ABTS method, and 0.063 ± 0.01 mg/mL and 0.009 ± 0.008 mg/mL by the H_2_O_2_ method. For the FRAP technique, the EC_50_ was between 0.42 ± 0.02 mg/mL and 0.09 ± 0.01 mg/mL. Finally, the equivalent concentration of ascorbic acid ranged between 10.42 ± 0.03 mg AAs/mL for *M. piperita* and 7.25 ± 0.19 mg AAs/mL for *T. serpyllum*. As determined by multivariate analysis, antioxidant activities through the DPPH, ABTS, TAC, and FRAP were mainly influenced the major compounds of *M. pulegium* and *M. piperita* EOs. However, the H_2_O_2_ method showed a stronger positive correlation with major compounds of *T. zygis* EO.

**Conclusion:** The EOs derived from *M. piperita*, *M. pulegium*, *T. serpyllum*, and *T. zygi*s species might be exploited as a natural source for antioxidant activity.

## 1. Introduction

Thanks to the use of various food additives during the last few years, the agricultural and food industries have undergone a significant revolution. While higher standards for sanitary and salubrity have been mandated, consumers are becoming more demanding when it comes to the variety and choice of agricultural and food products [1].

Food antioxidants are chemical compounds that are frequently manufactured. Although they have been shown to defend against free radicals and reactive oxygen species (ROS), they may also have negative impacts on consumer health [2]. Note that, a free radical is known as a chemical entity that possesses one or more unpaired electrons produced in the body during metabolism. When present in excess, harmful consequences might occur [3].

The inactivation of ROS or prevention of their cellular production by antioxidants and free radical scavengers is seen as a viable strategy for lowering the risk of diseases like cancers, cardiovascular disease, inflammations, and the aging process [4–6]. For example, sodium benzoate can cause skin reactions (eczema) and respiratory problems, and butylated hydroxyanisole (BHA), butylated hydroxytoluene (BHT), and tert-butylhydroquinone (TBHQ) are carcinogens while having strong antioxidant properties [7]. Therefore, replacing these synthetic additives with safe and nontoxic natural antioxidants is crucial.

Essential oils (EOs) are delicate, fragrant, and volatile liquids derived from diverse plant sections as secondary metabolites. Secondary metabolites perform critical ecological and biological functions, as well as being crucial for plant defense due to their antioxidative characteristics [8]. For ages, EOs have been widely used in the medicinal, agricultural, sanitary, and cosmetic sectors, as well as in cuisine as spices or herbs [9].

On the other hand, EOs have a high potential for application as food preservatives due to their ability to minimize oxidative reactions that occur during food handling, processing, and storage [10]. Furthermore, natural antioxidants such as phenolic compounds, flavonoids, and other phytochemicals can operate as free radical scavengers [11]. They also help to postpone lipid oxidation and increase consumer acceptability of food products [12]. Many studies have found that eating foods containing flavonoids on a daily basis may reduce the risk of some malignancies, such as colon, pancreatic, and breast cancer [13].

Among the most economically significant genera having aromatic and medicinal properties, the *Thymus* and the *Mentha* species belonging to the Lamiaceae family [14] are the most extensively utilized in terms of health and medicinal purposes, mostly because of menthol, menthone, thymol, and carvacrol [15].

The objective of this study was to analyze the aroma profile and antioxidant activity using five different methods, of two *Mentha* species (*Mentha piperita* and *Mentha pulegium*) and two *Thymus* species (*Thymus serpyllum* and *Thymus zygis*) EOs from Morocco. The correlation between the antioxidant activity and the major components of the four oils was also examined using a multivariate analysis.

To the best of our knowledge, this research is the first of its kind to assess these EOs in such detail. It not only evaluates their antioxidant activities using the five methods mentioned but also delves into the correlations between antioxidant activity and their key components. The findings of this study may represent an important step toward investigating the potential applications of these EOs as antioxidants in food products.

## 2. Materials and Methods

### 2.1. Plant Material

The *M. piperita*, *M. pulegium*, *T. serpyllum*, and *T. zygis* plants were cultivated and collected from Timezgana commune located in Taounate Province, North Center of Morocco (34°33′ 02.7″ N 4°40′ 49.3″ W) in April and May 2021. These species were identified by Professor Badr Satrani, botanist at the Forestry Research Center—Rabat (FRC-Rabat), Morocco. Each plant's voucher specimen was placed at the herbarium of Morocco's National Agency of Medicinal and Aromatic Plants.

### 2.2. Extraction of the EOs

All of aerial parts of the plants (with the branches) were dried under shade at ambient temperature at an average of 35 ± 4°C) by dispersing the herbs on paper for 72 h (shade drying).

Hydrodistillation of each plant's aerial parts (stems, leaves, and flowers) was carried out using a Clevenger-type apparatus for 2 h and 30 min. The EOs were then kept in amber-colored vials at 4°C until usage.

### 2.3. Yield Calculation

The EO yield was calculated in relation to the dry matter of the plant (calculated after putting a certain amount of the plant in the pond, evaporation of the water, and stabilization of the plant's weight) using the following equation [16]:

(1) $$\begin{array}{c} Y\left(\%\right)=\frac{\text{mEO}}{\text{mD}}\times 100\%, \end{array}$$

where mEO = mass of EO (g), mD = mass of dry plant material (g), and *Y* = yield of EO (%).

### 2.4. The Identification of EO Chemical Compositions

The components of *M. piperita*, *M. pulegium*, *T. serpyllum*, and *T. zygis* EOs were identified using a Shimadzu gas chromatography (Trace GC Ultra, Hewlett-Packard, HP 6890) coupled with a mass spectrometry (MS, HP 5973) (Tokyo, Japan) supplied with HP-5MS column (60 m × 0.32 mm × 0.25 *μ*m). The following requirements were made for the GC-MS analysis: At a flow rate of 1.0 mL/min, helium was employed as the carrier gas; the column temperature was planned to rise from 40°C to 280°C at a rate of 5°C/min; and at a temperature of 220°C, a sample volume of 1 *μ*L was injected in a split mode. Before attempting to inject the sampling port, the samples of EOs were diluted (1/20 v/v) in hexane. Quantitative analysis was performed by HP ChemStation software, and the identification of the components based on their Kovats retention index (KI) calculated against n-alkanes (C8-C23) series was verified by comparing their mass spectral fractionation with NIST MS Search database 2012 and by Adams terpenes library [17].

### 2.5. Antioxidant Activity

#### 2.5.1. 2,2-Diphenyl-1-picrylhydrazyl (DPPH) Assay

The free radical scavenging activity of the two EOs was evaluated by assessing their capacity to scavenge DPPH stable radicals. The DPPH test was carried out accurately as specified by [18] with a few modifications. The DPPH solution was prepared by dissolving 0.005 g of DPPH in 200 mL of absolute ethanol. Then, 25 *μ*L of the sample at various concentrations was mixed with 825 *μ*L of DPPH. After being agitated, the tubes were placed in the dark at room temperature for 1 h.

The reading was performed using an absorbance measurement at 517 nm. Moreover, the results obtained for each tested EO were compared to those obtained for BHT, which was used as a control. The antiradical activity was calculated using the following equation:

(2) $$\begin{array}{c} I\;\left(\%\right)\left[\text{DPPH}\right]=\left[\frac{\text{Abs control}-\text{Abs sample}}{\text{Abs}}\text{control}\right]\times 100, \end{array}$$

where Abs control is the absorbance of the control (including all reagents except the EOs for testing) and Abs sample is the absorbance of tested EOs.

IC_50_ values, which reflected the EO concentration that triggered 50% scavenging, were determined graphically from a plot of I (%) vs concentration.

#### 2.5.2. The Cation Radical 2,2′-Azino-bis-(3-ethylbenzothiazoline-6-sulfonic Acid) (ABTS) Assay

The ability of an antioxidant agent to inhibit the radical cation ABTS was determined using a modified version of the method described by [19]. This method is based on transforming a stable radical cation, ABTS^+^ into ABTS in the presence of antioxidants.

The radical cation ABTS+ was regenerated by reacting an ABTS (7 mM) solution with 2.5 mM of potassium persulfate, and the mixture is kept in the dark at room temperature for 16 h before use. The mixture was then diluted with ethanol to provide a spectrophotometric absorbance of 0.70–734 nm. Subsequently, 50 *μ*L of the sample at various concentrations was mixed with 825 *μ*L of ABTS^+^.

After 6 min, the absorbance was measured at 734 nm, and the results for each tested EO were compared to those obtained for gallic acid, which was used as a control. The antiradical activity was calculated using the following equation:

(3) $$\begin{array}{c} I\;\left(\%\right)\;\left[\text{ABTS}\right]=\left[\frac{\text{Abs control}-\text{Abs sample}}{\text{Abs}}\text{control}\right]\times 100. \end{array}$$

The IC_50_ values were determined graphically using linear regression.

#### 2.5.3. Hydrogen Peroxide Scavenging Capacity (H_2_O_2_) Assay

The capacity of the four EOs to scavenge hydrogen peroxide was assessed using the technique of [20] with slight modifications. In phosphate buffer (50 mM, pH = 7.4), a solution of hydrogen peroxide (40 mM) was produced. A total of 100 *μ*L of each EO concentration was mixed with a hydrogen peroxide solution (0.6 mL and 40 mM), one mL of phosphate buffer (50 mM, pH = 7.4), and 1650 *μ*L of distilled water. After 20 min, the absorbance of hydrogen peroxide at 230 nm was measured in comparison with a blank solution containing the phosphate buffer but no hydrogen peroxide.

The hydrogen peroxide inhibition percentage was calculated as follows:

(4) $$\begin{array}{c} I\;\left(\%\right)\;\left[H2O2\right]=\left[\frac{\text{Abs control}-\text{Abs sample}}{\text{Abs}}\text{control}\right]\times 100. \end{array}$$

IC_50_ values were determined graphically from a plot of % scavenging vs concentration.

#### 2.5.4. Ferric Reducing Antioxidant Power (FRAP) Assay

The ability of an antioxidant compound to reduce iron was determined using the method described by [21], with some modifications. This method is based on the capacity of EOs to reduce the ferric ion (Fe^3+^) present in the potassium ferricyanide complex to ferrous ion (Fe^2+^). A total of 50 *μ*L of EOs at various concentrations were mixed with 200 *μ*L of a tampon phosphate solution 0.2 M (pH 6.6) and 200 *μ*L of a potassium ferricyanide solution (1%). After 20 min in a water bath of 50°C, 200 *μ*L of trichloroacetic acid (10%), 200 *μ*L of distillate water, and 120 *μ*L of FeCl3 (0.1%) were added.

The absorbance of the reactionary medium was measured at 700 nm. An increase in absorbance corresponds to an increase in the reductive power of the tested sample. The positive control was represented by a solution of a standard antioxidant: ascorbic acid, at the same conditions as the samples. IC_50_ values were determined graphically from a plot of % scavenging vs concentration.

#### 2.5.5. Total Antioxidant Capacity (TAC)

The phosphomolybdate test was carried out using the method described by [22], with a few changes. This approach was based on converting molybdates to molybdenum in the presence of an antioxidant, which produces a green complex that absorbs at 700 nm.

The phosphomolybdate reagent was created by combining sulfuric acid (25 mL in 225 mL of distillate water), sodium phosphate (3.28 g in 250 mL of distillate water), and ammonium molybdate (3.7 g in 250 mL of distillate water). Briefly, 100 *μ*L of sample and standard was mixed with 1 mL of phosphomolybdate reagent. After 1°h 30 of incubation at 96°C, the absorbance was measured at 700 nm.

A typical blank solution contained 1 mL of reagent solution and the appropriate amount of the same solvent used for the sample was incubated with the other samples under the same conditions. The antioxidant capacity was calculated using an established calibration range with ascorbic acid and was expressed in milligrams of ascorbic acid equivalent per milliliter of EO (mg Eq.AAs/mL).

### 2.6. Statistical Analysis

All tests were carried out in triplicate. The three measurements for each data point in each independent repeat were first utilized to determine the average values and standard deviations (SDs) shown in the results, as well as to perform statistical analysis. The data were analyzed using computer software Excel 2010 and represented as mean ± SD.

### 2.7. Principal Component Analysis (PCA), Hierarchical Cluster Analysis (HCA), and Study of Correlations Between Major Compounds and Antioxidant Activity

#### 2.7.1. Explained Variability

To determine the number of components to retain, we will adopt Kaiser's criterion, which states that in a normalized PCA, and we retain the components whose eigenvalues are greater than 1. The table of explained variability (Table 1) showed that the first three components have eigenvalues greater than 1 and explained 100% variability, while the first two components explain 79% variability.

## 3. Results and Discussion

### 3.1. The EO Yields and Chemical Composition

The EO average yields obtained in this investigation are shown in Table 2, which showed that *M. piperita*, *M. pulegium*, *T. serpyllum*, and *T. zygis* EO yields were 1.9%, 2.4%, 0.8%, and 2.7%, respectively. These results were higher than those reported in other studies (Table 2).

These variations in yields could be attributed to various factors, including climatological conditions, genetic factors, geographic distribution, collection period, and extraction method [34]. As reported by [35, 36], these changes may also be caused by the plant's maturity, its age, and interaction with the environment (soil, climate, etc.).

**Figure 1:**
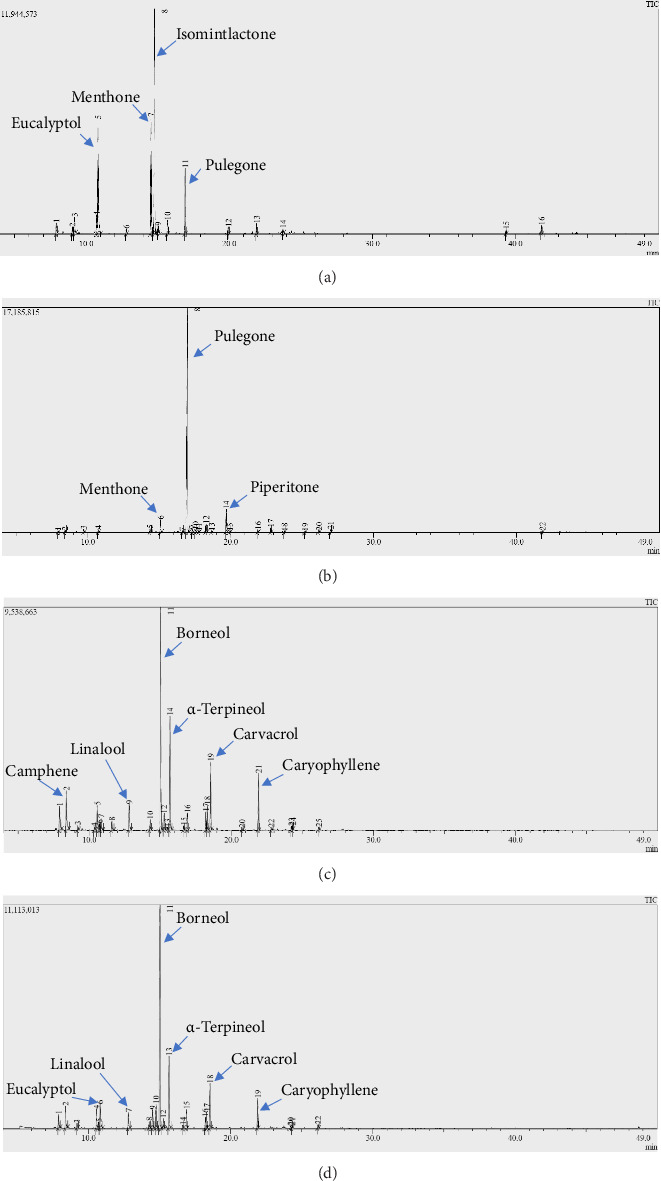
Chromatograms of essential oils: (a) *M. piperita*; (b) *M. pulegium*; (c) *T. zygis*; (d) *T. serpyllum*.

The results of chromatographic analysis of *M. piperita*, *M. pulegium*, *T. serpyllum*, and *T. zygis* EOs are presented in Table 3 and Figure 1. They revealed that four volatile substances made up 82.44% of *M. piperita* EO. Three major constituents were detected in the *M. pulegium* EO, accounting for 86.07% of the total EO. *T. serpyllum* EO analysis found 10 distinct components that account for 84.16% of its content. For *T. zygis* EO, nine volatile components were discovered, representing 83.46% of this EO.

The composition of EO assessed in our investigation revealed that the main constituents were as follows: isomintlactone (35.55%), menthone (18.48%), eucalyptol (18.04%), and pulegone (10.37%). On the other part, [37] found menthol (59.73%), followed by isomenthone (18.45%) and methyl acetate (6.02%) in Brazilian EO. In the same country but in a different region, de Sousa, de Morais, and Ferreira [30] obtained carvone D (49.27%) and limonene (37.18%) as major components in *M. piperita* EO. Menthol (49.89%), menthone (20.84%), isomenthone (7.25%), 1,8-cineole (6.73%), and ciscarane (4.99%) characterized the Algerian oil [38]. In India, [39] obtained menthol (34.82%), carvone (19.54%), and menthone (9.10%). Reference [40] reported that menthol (4.30%), caryophyllene (5.50%), and 1,8-cineole (62.16%) were the major components of Korean *M. piperita* EO. Iranian and Saudi Arabian *M. piperita* EOs possessed almost identical components: menthol (36.9%), menthone (28.8%), menthyl acetate (4.54%), and 1,8-cineole (3.75%) for the Iranian EO by [41] and menthol (36.02%), menthone (24.56%), menthyl acetate (8.95%), and menthofuran (6.88%) for the Saudi Arabian EO by Desam et al. [42].

The EO of *M. pulegium* was composed of 22 components. The main components were pulegone (74.04%), piperitone (7.94%), and menthone (4.09%). It has been observed that the presence of pulegone as a major constituent of this oil indicated that it is of the pulegone chemotype. These findings were similar to the majority of previous studies on *M. pulegium* EO, albeit at different percentages [43–46]. Other authors have found other components as major components of *M. pulegium* EO: pulegone (75.48%), carvone (6.66%), and dihydrocarvone (4.64%) by Chraibi et al. [47]; isomenthone (13.4%) by [48]; menthone (21.16%) and pulegone (40.98%) by Bouyahya al. [49]; pulegone (69.8%) and piperitenone (3.1%) by [50]; and piperitone (38.00%), piperitenone (33.0%), and *α*-terpineol (4.7%) by [51].

Twenty-two components representing 100% of the total amount of *T. serpyllum* EO have been identified. The main constituents were borneol (37.87%), *α*-terpineol (12.23%), carvacrol (7.58%), caryophyllene (5.06%), isomintlactone (4.13%), eucalyptol (4.06%), camphene (3.72%), o-cymene (3.26%), 1,3-dimethyladamantane (3.14%), and pulegone (3.11%). Except for a few common compounds, this olfactory profile differs from that of many other authors. In fact, [52] obtained *γ*-terpinene (13.70%), thymol (13.70%), carvacrol (13.60%), *β*-bisabolene (8.50%), *p*-cymene (6.30%), farnesol (3.60%), elemol (3.60%), and ethyl carvacrol (3.10%). The EO studied by [53] was mostly composed of thymol (31.29 ± 1.13%), *α*-terpinene (14.58 ± 0.09%), carvacrol (5.11 ± 0.08%), and *α*-pinene (3.28 ± 0.03%). The main components obtained by [25] were carvacrol (35%–56%), thymol (11%–29%), *γ*-terpinene (14%–17%), and *p*-cymene (11%–15%). Reference [54] found carvacrol (66%) and *γ*-terpinene (11.5%) in Algerian EO. To compare with the EOs of Moroccan plants, [55] discovered linalyl (52.20%), (E)-nerolidol (15.10%), geranyl (5.00%), *α*-terpineol (4.30%), and terpinyl (3.40%) for plants harvested in the south and southeast of Morocco. Ouedrhiri et al. [24] obtained *p*-cymene (36.15%), *γ*-terpinene (18.31%), thymol (17.29%), and linalool (4.51%) from the EO of the plant harvested in Taounate (the same region as our harvest). Almost all studies on *T. serpyllum* showed that carvacrol and thymol are major components of their EOs [56–10].

In terms of *T. zygis* EO's chemical composition, the main components were borneol (30.99%), *α*-terpineol (15.89%), carvacrol (9.47%), caryophyllene (7.89%), camphene (5.45%), linalool (3.60%), thymol (3.54%), *o*-cymene (3.41%), and *α*-pinene (3.22). In accordance with this research, [60] reported that the major components of *T. zygis* EO cultured in Portugal were *p*-cymene (22.0%), thymol (19.5%), carvacrol (16.3%), *γ*-terpinene (7.4%), linalool (5.5%), and borneol (3.4%). Also, [61, 62] found thymol (68.1%), *p*-cymene (11.2%), *γ*-terpinene (4.8%), and carvacrol (3.5%) as major components in Spanish EO and carvacrol (16.07%–74.33%), thymol (1.47%–32.46%), *p*-cymene (6.97%–40.26%), and *γ*-terpinene (2.68%–22%) in Moroccan EO. However, this composition differed from that of the EO of *T. zygis* studied in Saudi Arabia and South Africa, where the major components were linalool (39.7%), terpinen-4-ol (11.7%), *β*-myrcene (8.6%), and *γ*-terpinene (7.6%) reported by [63], and *p*-thymol (46.393%) and *p*-cymene (22.154%) reported by [64]. References [32, 65–68] also revealed a chemical composition that differed from ours.

The quantitative and qualitative differences in the composition of EOs may be related to the origin of the plant, climate and geographical circumstances, and genetic diversity [69–70]. This variance might also be attributable to environmental factors, agronomic conditions, drying method, oil extraction methodology, and harvesting season [38, 71, 72].

### 3.2. Antioxidant Activity

Antioxidant activity of the obtained EOs was determined by using five different previously described methods (DPPH, ABTS, H_2_O_2_, FRAP, and TAC), because a single technique will offer basic information regarding antioxidant characteristics, but a combination of methods will characterize the sample's antioxidant qualities in greater depth [73].

#### 3.2.1. DPPH Scavenging Activity

The IC_50_ value is defined as the concentration of plant EO necessary to scavenge half of the total DPPH radicals (50%) present. Scavenging of free radical DPPH was an approach utilized to investigate the antioxidant activity of the two *Mentha* and the two *Thymus* species. The *T. zygis* EO with IC_50_ = 0.42 ± 0.07 mg/mL was the most active, followed by *T. serpyllum*, *M. piperita*, and *M. pulegium*, with an IC_50_ of 1.39 ± 0.34 mg/mL, 2.99 ± 0.20 mg/mL, and 3.24 ± 0.20 mg/mL, respectively. The difference in antioxidant capacity of the four EOs might be attributed to different chemical profiles, with different functional groups, polarity, and chemical behavior [74].

The inhibitory concentration 50 determined for *M. piperita* (IC_50_ = 2.99 ± 0.20 mg/mL) was lower than that reported by other authors as an example [75], who determined an IC_50_ of (IC_50_ = 7.81 ± 0.23 mg/mL) for Iranian EO. Meanwhile, the Algerian EO of *M. piperita* studied by [76] showed a higher activity of radical scavenging than our result, which is on the order of 17.0 *μ*g/mL. Other studies, such as those of [77] and Singh et al. [29], have demonstrated the significant antioxidant activity of Libyan *M. piperita* EO ((IC_50_ = 0.86 mg/mL) and (IC_50_ = 0.015 ± 0.009 mg/mL)), respectively. The differences in the antioxidant capacity of peppermint oil can be explained by the complex and changeable chemistry of this EO, which varies according on the climate, cultivar, and geographical location [29].

The EO of *M. pulegium* had an IC_50_ of IC_50_ = 3.24 ± 0.20 mg/mL. This activity was lower than that of the other three EOs. The EOs of *M. pulegium* studied by Bouyahya et al. [49] from Morocco [78], from Tunisia, and [76] from Algeria showed higher radical scavenging activities than our result, which were in the range of IC_50_ = 0.32 ± 0.002 mg/mL, IC_50_ = 0.14 mg/mL, and IC_50_ = 0.025 mg/mL, respectively. On the opposite, several researchers had reported that this EO has a lower antioxidant activity (IC_50_ = 14.73 ± 0.15 mg/mL) by [79] from Iran and IC_50_ = 6.2 ± 0.2 mg/mL by [1] from Portugal. The composition of *M. pulegium's* EO may be the source of its antioxidative activity; this can be mainly attributed to the concentration of the main components: pulegone, menthone, and piperitenone, or refers to the interaction of these three elements either antagonistically, synergistically, or both. In this regard, the most powerful components in this EO were monoterpene ketones [80]. According to de Sousa, de Morais, and Ferreira [30], the antioxidant activity of Mentha species is related to a higher piperitenone concentration. The antioxidant potential of *M. pulegium* EO was confirmed by [81], who reported that pulegone and menthone were known to have significant antioxidant activity. It is well known that the antioxidant activity of EOs is heavily dependent on their chemical composition, with special attention paid to the oxygenated compounds found in them [74].

*T. serpyllum* EO had demonstrated significant activity in radical scavenging (IC_50_ = 1.39 ± 0.34 mg/mL), and this is due to the unique chemical profile of this oil, which is devoid of oxygenated monoterpenes [82], whose have well-known antioxidant effects such as thymol, carvacrol, *γ*-terpinene, and linalool. According to the literature, the antioxidant activity of *T. serpyllum* EO as measured by the DPPH test appeared to be significantly higher than that of other authors' studies: IC_50_ = 34.8 mg/mL by [83] and an IC_50_ higher than 30,000 *μ*g/mL by [55]. Meanwhile, the oils tested by [56, 59, 10] and [83] were more active than our EO, with an IC_50_ = 0.96 ± 0.05 mg/mL, IC_50_ = 384.60 *μ*g/mL, IC_50_ = 34 µg/mL, and IC_50_ = 0.96 *μ*g/mL, respectively.

*T. zygis* EO was the most efficient in scavenging DPPH radicals, with IC_50_ = 0.42 ± 0.07 mg/mL, which, however, was always superior to the positive control (BHT), with IC_50_ = 0.021 ± 0.001 mg/mL. This is owing to the oil's distinct chemical composition and maybe linked to the synergistic and cumulative effects of its contents. The IC_50_ values obtained in our study were comparable to those published by other researchers [84, 85] and much higher than those reported by [33, 86]. [32] also discovered a less effective antioxidative activity, as evidenced by a higher IC_50_ value (IC_50_ = 3.7 ± 1.6 mg/mL).

#### 3.2.2. The Cation Radical ABTS

The results of the ABTS test supported the results of the DPPH test by classifying *T. zygis* as the most active EO with an IC_50_ of 0.40 ± 0.04 mg/mL and *M. pulegium* as the EO with the lowest antioxidant activity, with an IC_50_ of 1.02 ± 0.21 mg/mL. The other EOs had an IC_50_ value of 0.44 ± 0.7 mg/mL for *T. serpyllum* and 0.96 ± 0.29 mg/mL for *M. piperita*. The gallic acid had the highest activity, with an IC_50_ of 0.019 ± 0.001 mg/mL.

To the best of our knowledge, the number of published studies on the antioxidant activity of *M. piperita, M. pulegium*, and *T. zygis* EOs utilizing the ABTS technique is rather modest; however, it has never been tested on the EO of *T. serpyllum*. Previous research has revealed that both *Mentha* species and *T. zygis* EOs have varied antioxidant activities: an IC_50_ varying from 1.41 ± 0.01 to 6.59 ± 0.02 *μ*g/mL for Serbian *M. piperita* EO [87], an IC50 = 155 *μ*g/mL for Tunisian EO [78], IC_50_ = 57.4 ± 1.9 *μ*g/mL [88], an IC_50_ = 5.72 ± 0.060 mg/mL [74] for Algerian *M. pulegium* EO, and finally an IC_50_ = 2.07 mg/mL for *T. zygis* [85].

#### 3.2.3. Hydrogen Peroxide Scavenging Capacity (H_2_O_2_)

The antioxidant activity of EOs of *M. piperita*, *M. pulegium*, *T. serpyllum*, and *T. zygis* was evaluated by hydrogen peroxide scavenging capacity method (H_2_O_2_). The results of the H_2_O_2_ test also support the results of the DPPH and ABTS tests. *T. zygis* and *T. serpyllum* showed significant antioxidant activity compared to the ascorbic acid with an IC_50_ of 0.063 ± 0.01 mg/mL, 0.05 ± 0.004 mg/mL, and 0.023 ± 0.03 mg/mL, respectively, while *M. piperita* and *M. pulegium* significantly reduced H_2_O_2_ with an IC_50_ = 0.013 ± 0.003 mg/mL and IC_50_ = 0.009 ± 0.008 mg/mL, respectively. The H_2_O_2_ test findings were encouraging when compared to those obtained with ascorbic acid, which was employed as a control.

To the best of our knowledge, according to the literature, the antioxidant capacity of *Thymus* species (including *T. serpyllum* and *T. zygis*) and *M. pulegium* has not been investigated using this approach. In contrast, one H_2_O_2_ activity has been reported by [89] for *M. piperita* (IC_50_ = 34.81 ± 0.01 *μ*g/mL), which was higher than the value obtained in our study.

#### 3.2.4. FRAP

Table 4 displayed the findings of investigating the antioxidant activity of *M. piperita*, *M. pulegium*, *T. serpyllum*, and *T. zygis* EOs by the iron reduction method (FRAP). The iron-reducing activity of *M. pulegium* EO was higher than that of the other EOs, followed by *T. serpyllum* EO, *M. piperita* EO, and *T. zygis* EO, respectively. The iron-reducing activity of all EOs was higher than that of ascorbic acid, which was used as a control with a best iron reduction. The antioxidant activity of the four EOs was quantified by estimating the effective concentration (EC_50_).

From these results, we can deduce that the EC_50_ to reduce 50% of the Fe^3+^ ions were 0.42 ± 0.02 mg/mL, 0.161 ± 0.002 mg/mL, 0.101 ± 0.01, 0.09 ± 0.01 mg/mL, and 0.035 ± 0.010 mg/mL, for *M. pulegium*, *T. serpyllum*, *M. piperita*, *T. zygis*, and ascorbic acid, respectively. These results were lower than those discovered by [75, 87] for Serbian and Iranian *M. piperita* [74, 78, 53]; for Tunisian, Moroccan, and Algerian *M. pulegium*; and [54, 59] for *T. serpyllum*. On the other hand, [33] discovered an EC_50_ of 2.46 ± 0.01 *μ*g/mL for *T. zygis*, which was lower than the one found in our study.

There is a link between the chemical composition and the reported antioxidant activity, particularly with the high quantities of some components like carvacrol, menthone and pulegone…, which have a strong antioxidant potential. Indeed, multiple investigations have shown that EOs containing phenolic chemotypes have better antioxidant potential [84, 90]. Due to their redox characteristics, phenols act as reducing agents, hydrogen donors, and single oxygen donors. Other minor components, however, can interact directly, synergistically, antagonistically or both to produce a combination with greater action. Furthermore, the antioxidant activity of the bulk of the components evaluated independently gives lower findings when compared to the activity of the EO as a whole [91].

#### 3.2.5. TAC

TAC assessed by the phosphomolybdate method for the four EOs ranged from 10.42 ± 0.03 mg AAs/mL in *M. piperita* to 7.25 ± 0.19 mg AAs/mL in *T. serpyllum*. The maximum concentrations of the other EOs were 9.94 ± 0.21 mg AAs/mL in *M. pulegium* and 8.36 ± 0.42 mg AAs/mL in *T. zygis*. The results of all EOs were dose-dependent, considering antioxidant activity increased with concentration.

The phosphomolybdate reduction capacity of *M. piperita*'s EO was (10.42 ± 0.03 mg AAs/mL), and this value appeared to be greater than that provided by [92] for the same species from Serbia. The phosphomolybdate test on *M. pulegium*'s EO (9.94 ± 0.21 mg AAs/mL) confirmed its potent antioxidant activity. As a result, our EO has a much higher activity than [93] of the same species found in Algeria, which is in the range of 159 *μ*g AAs/mL. Similarly, the research of [74] on the chemical composition and biological activity of *M. pulegium*'s EO yielded a value in the order of 109 *μ*g AAs/mL that was significantly lower than ours.

To the best of our knowledge, the phosphomolybdate test has never been performed on the EOs of *T. serpyllum* and *T. zygis*. As a result, we will compare these EOs to those of other species of the same genre. In this regard, a study conducted by [93] with the goal of studying the chemical composition and antioxidative and antimicrobial activities of EOs from Algerian Lamiaceae species, and it was discovered that the antioxidant capacity of *T. algeriensis* and *T. vulgaris* EOs was evaluated using the phosphomolybdate method, showing values in the range of 220 *μ*g AAs/mL and 107 *μ*g AAs/mL, respectively, which were lower than our result for *T. serpyllum* (7.25 ± 0.19 mg AAs/mL) and *T. zygis* (8.36 ± 0.42 mg AAs/mL). Other studies on the *Thymus* species had also been conducted. References [94, 95] discovered an antioxidant activity of *T. vulgaris* in the order of 90 *μ*g AAs/mL and an antioxidant activity of *T. spathulifolius* in the order of 502 *μ*g AAs/mL, respectively, which were still less than our values.

### 3.3. Multivariate Analysis

#### 3.3.1. PCA

##### 3.3.1.1. Study of Variables

**Figure 2:**
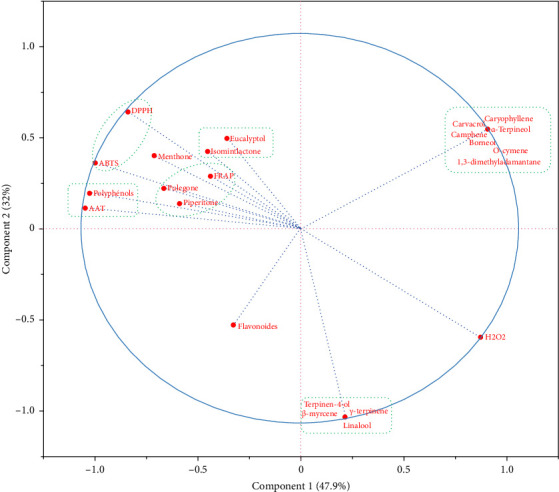
Graph of the distribution of the variables on the first and second principal components.

The loading plot analysis revealed several significant findings (Figure 2). Firstly, the compounds borneol, *α*-terpineol, carvacrol, caryophyllene, camphene, 1,3-dimethyladamantane, and *o*-cymene exhibited positive correlations with each other. Secondly, the compounds *β*-myrcene, linalool, terpen-4-ol, and *γ*-terpinene demonstrated intercorrelations. Additionally, isomintlactone and eucalyptol were found to be positively correlated. Furthermore, a positive correlation was observed between total polyphenols and TAC. Moreover, FRAP antioxidant activity displayed correlations with both DPPH and piperitone. Notably, the antioxidant activities of DPPH and ABTS were positively correlated. The Pearson correlation test (*p value* < 0.05) statistically confirmed all identified correlations.

The loading plot analysis revealed several significant findings. Firstly, the compounds borneol, *α*-terpineol, carvacrol, caryophyllene, camphene, 1,3-dimethyladamantane, and *o*-cymene exhibited positive correlations with each other. Secondly, the compounds *β*-myrcene, linalool, terpen-4-ol, and *γ*-terpinene demonstrated intercorrelations. Additionally, isomintlactone and eucalyptol were found to be positively correlated. Furthermore, a positive correlation was observed between total polyphenols and TAC. Moreover, FRAP antioxidant activity displayed correlations with both DPPH and piperitone. Notably, the antioxidant activities of DPPH and ABTS were positively correlated. The Pearson correlation test (*p value* < 0.05) statistically confirmed all identified correlations.

The positive correlation between polyphenols and antioxidant activity was confirmed in vivo by the literature. Reference [96] reported that polyphenols exert cytoprotection effect by reducing the formation of oxygen free radicals. The same correlation was observed by [97].

##### 3.3.1.2. Score Plot

**Figure 3:**
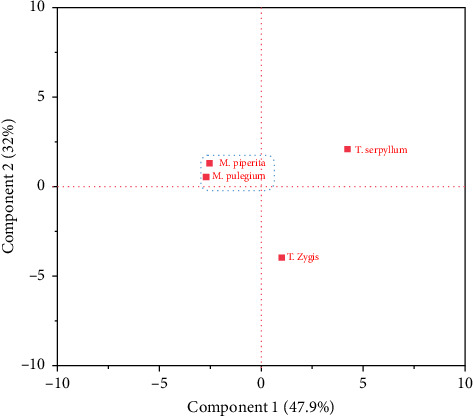
Individual graph of the four essential oils.

The data presented in Figure 3 reveals interesting insights about the chemical composition and antioxidant activities of four different species: *M. pulegium*, *M. piperita*, *T. serpyllum*, and *T. zygis*. Upon analyzing the graph, it becomes evident that the first two species, *M. pulegium* and *M. piperita*, exhibited striking similarities in both their chemical composition and antioxidant activities. This suggests that these two species share comparable properties in terms of the compounds they contain and their ability to combat oxidative stress.

In contrast, the score plot (Figure 3) highlights a noticeable distinction between *M. pulegium* and *M. piperita*, and the other two species, *T. serpyllum* and *T. zygis*. The chemical composition and antioxidant activities of *T. serpyllum* and *T. zygis* differed considerably from those of *M. pulegium* and *M. piperita*, indicating unique properties exclusive to these two species.

##### 3.3.1.3. Biplot Scores/Loadings

**Figure 4:**
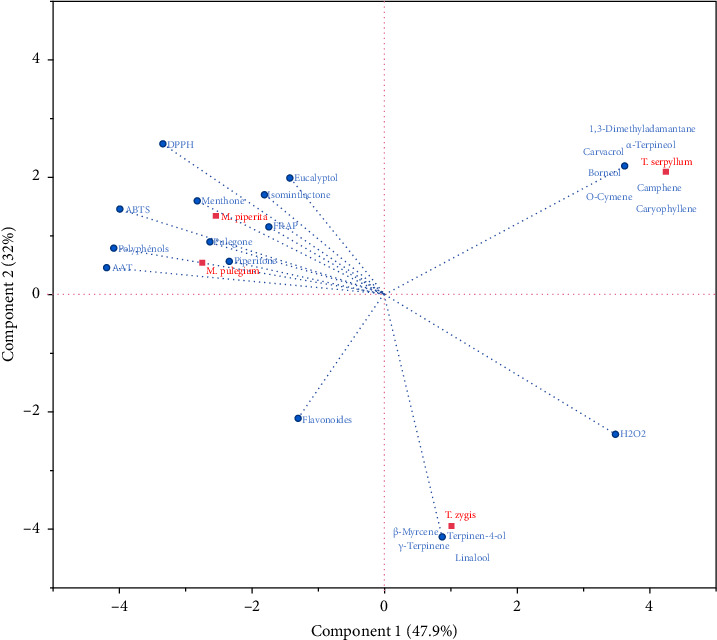
Graph of distribution of variables and individuals on the first and second principal components.

The biplot presented in Figure 4 highlighted that the antioxidant activities assessed using the DPPH, FRAP, ABTS, and TAC methods were predominantly associated with *M. pulegium* and *M. piperita* species. This connection can be attributed to their higher concentrations of highly active major compounds. Conversely, the influence on H_2_O_2_ activity was more pronounced in the case of *T. serpyllum* and *T. zygis* species. Furthermore, in terms of total phenolic content, the two species of *Mentha* exhibited the highest richness.

#### 3.3.2. HCA

The results obtained by PCA were confirmed by the HCA method as shown in the graph.

**Figure 5:**
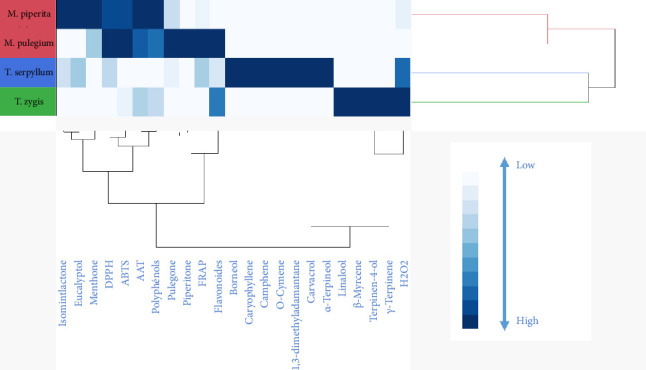
Hierarchical classification of individuals according to their major compounds and their antioxidant activities.

Figure 5 presents a comprehensive analysis of the antioxidant activities using various methods such as DPPH, ABTS, TAC, FRAP, and H_2_O_2_. The data reveal an interesting correlation between the antioxidant activities and the major compounds found in the two species, *M. pulegium* and *M. piperita*.

Specifically, when assessing the antioxidant activities through the DPPH, ABTS, TAC, and FRAP methods, it becomes evident that these activities were primarily influenced by the major compounds present in *M. pulegium* and *M. piperita*. This indicates that these two species possess significant antioxidant potential due to the presence of specific compounds that contribute to their observed activities in these methods.

In contrast, the H_2_O_2_ method demonstrated a closer association with the major compounds found in *T. zygis* EO. This suggests that the antioxidant activity measured through the H_2_O_2_ method is more influenced by the specific compounds present in *T. zygis*. These compounds likely play a crucial role in conferring antioxidant properties to *T. zygis*, distinguishing it from *M. pulegium* and *M. piperita* in terms of this particular method.

## 4. Conclusion

Based on our results, the EOs of *M. piperita*, *M. pulegium*, *T. serpyllum*, and *T. zygis* were found to be very rich in some components (e.g., menthol for *M. piperita*; pulegone and menthone for *M. pulegium*; thymol and carvacrol for *T. serpyllum* and *T. zygis*), which remain the main contributors to the biological activities of these oils. The evaluated EOs demonstrated exceptional antioxidant activity using all five techniques. For the correlations between the major compounds of the four oils and their antioxidant effect using PCA and HCA, the two *Mentha* species could be classified in the same group because they contained the main constituents in significant relation with the antioxidant activities by the DPPH, ABTS, TAC, and FRAP methods. The other two classes contained the other two species *T. serpyllum* and *T. zygis* with a relationship with the H_2_O_2_ method. According to the results of this investigation, the selected native species of *M. piperita*, *M. pulegium*, *T. serpyllum*, and *T. zygis* are significant natural sources with extraordinary antioxidant activity, and their use in food applications is strongly recommended. Future research on formulation studies and the potential toxicity of examined EOs is needed for safety concerns, including in vitro and in silico studies using bioactive molecules [98–99].

## Figures and Tables

**Table 1:** The eigenvalues of the components, the percentages of explained variability, and the cumulative percentages of the variables on the first three components.

Number	Eigenvalue	Percentage	Cumulative percentage
1	11.0074	47.858	47.858
2	7.3700	32.043	79.902
3	4.6226	20.098	100.000

**Table 2:** Average yields of EOs of *M. piperita, M. pulegium, T. serpyllum*, and *T. zygis*.

EOs	Yields (%)	Yields reported in the literature (%)
*M. piperita*	1.9	0.93 [23]
		0.90 [24]
		0.40–1.4 [25]

*M. pulegium*	2.4	1.00 [26]
		1.93 [27]
		1.66 [28]

*T. serpyllum*	0.8	0.64 [29]
		0.10 [30]
		1.90 [31]

*T. zygis*	2.7	2.30 ± 0.70 [32]
		5.25 ± 0.01 [33]

**Table 3:** The chemical composition of *M. piperita*, *M. pulegium*, *T. serpyllum*, and *T. zygis* EOs.

Compounds	RI exp	RI ref	(%)			
			*M. piperita*	*M. pulegium*	*T. serpyllum*	*T. zygis*
*α*-Pinene	948	949	1.71	0.39	2.28	3.22
Camphene	943	948	—	—	3.72	5.45
3-Methylcyclohexanone	952	937	—	0.48	—	—
Sabinene (*β*-thujene)	897	920	1.13	—	—	—
*β*-Pinene	943	949	2.53	—	0.74	0.63
3-Octanone	979	985	—	0.84	—	—
*δ*-Carene	998	1002	—	—	—	0.44
*o*-Cymene	1042	1026	—	—	3.26	3.41
*D*-Limonene	1018	1029	2.90	0.94	0.98	0.89
Eucalyptol (1,8-cineole)	1059	1031	18.04	—	4.06	1.66
*γ*-Terpinene	998	1059	—	—	—	1.26
Linalool	1082	1085	0.78	—	2.67	3.60
Camphor	1121	1135	—	—	1.17	1.48
*α*-Terpinyl acetate	1350	1352	—	1.17	—	—
Menthone	1148	1157	18.48	4.09	3.11	—
Isomintlactone	1142	1219	35.55	—	4.13	—
Borneol	1138	1169	1.08	—	37.87	30.99
L-4-Terpineol	1137	1131	—	—	1.61	2.20
4,7,7-Trimethylbicyclo[4.1.0]heptan-3-ol, (1*α*,3*β*,4*α*,6*α*)-	1125	1127	—	—	—	0.36
*α*-Fenchol	1138	1100	2.15	—	—	—
*α*-Terpineol	1143	1143	—	—	12.23	15.89
1,6,6-Trimethyl-8-oxabicyclo[3.2.1]octan-2-one	1230	1229	—	0.30	—	—
Isoborneol formate	1275	1227	—	—	—	0.62
Bornyl formate	1277	1239	—	—	0.47	
Isothymol methyl ether	1231	1238	—	—	—	2.37
1,3-Dimethyladamantane	1290	1296	—	—	3.14	—
Pulegone	1212	1237	10.37	74.04	—	—
*α*-Elemene	1142	1177	—	0.90	—	—
Acid methyl ester	1372	1378	—	1.12	—	—
Piperitone	1190	1232	—	7.94	—	—
Fenchyl acetate	1277	1221	—	—	1.83	2.54
Acetaldehyde	1115	1068	—	2.68	—	—
Thymol	1262	1290	—	—	2.74	3.54
Carvacrol	1262	1299	—	—	7.58	9.47
Menthofuran	1368	1364	0.99	0.32	—	—
Copaene	1344	1378	—	—	—	0.31
Caryophyllene	1494	1466	1.65	0.76	5.06	7.89
*α*-Humulene	1494	1446	—	1.56	—	0.35
(+)-Mintlactone	1368	1368	0.75	0.31	—	—
*γ*-Cadinene	1435	1513	—	—	0.42	0.55
*δ*-Cadinene	1469	1514	—	—	0.37	0.54
Benzofuranone	1579	1539	—	0.28	—	—
Caryophyllene oxide	1368	1583	—	0.57	0.55	0.35
Humulene epoxide II	1518	1593	—	0.86	—	—
*p-*Cresol	1565	1071	0.68	—	—	—
*β*-Resorcylaldehyde	1650	1450	1.23	0.45	—	—
Monoterpene hydrocarbons			42.88	4.41	19.86	15.39
Oxygenated monoterpenes			14.35	75.28	50.81	49.36
Sesquiterpene hydrocarbons			1.65	4.65	6.40	9.99
Esters			—	2.29	—	—
Keto-alcohols			35.55	—	4.13	—
Ketones			0.75	9.85	—	—
Alcohols			3.61	—	16.51	22.05
Aldehydes			1.23	3.13	—	—
Total			100	100	100	100

*Note:* RI exp, retention index obtained; RI Ref, retention index of literature; monoterpene hydrocarbons, *α*-pinene, camphene, *β*-thujene, *β*-pinene, *δ*-carene, *o*-cymene, eucalyptol, *γ*-terpinene, menthone, 1,3-dimethyladamantane, fenchyl acetate, and menthofuran; oxygenated monoterpenes, *D*-limonene, camphor, borneol, 1,6,6-trimethyl-8-oxabicyclo[3.2.1]octan-2-one, isoborneol formate, bornyl formate, isothymol methyl ether, pulegone, thymol, and carvacrol; sesquiterpene hydrocarbons, *α*-elemene, copaene, caryophyllene, *α*-humulene, *γ*-cadinene, *δ*-cadinene, caryophyllene oxide, and humulene epoxide II; esters, *α*-terpinyl acetate, and acid methyl ester; keto-alcohols, isomintlactone; ketones, 3-methylcyclohexanone, 3-octanone, piperitone, mintlactone, and benzofuranone; alcohols, linalool, L-4-terpineol, 4,7,7-trimethylbicyclo[4.1.0]heptan-3-ol, (1*α*,3*β*,4*α*,6*α*)-, *α*-fenchol, *α*-terpineol, and *p*-cresol; aldehydes, acetaldehyde, and *β*-resorcylaldehyde.

**Table 4:** DPPH, ABTS, H_2_O_2_, and FRAP IC_50_ of *M. piperita*, *M. pulegium*, *T. serpyllum*, and *T. zygis* EOs and the ascorbic acid equivalence using the TAC assay.

Plants EO	DPPH (IC_50_) (mg/mL)	ABTS (IC_50_) (mg/mL)	H_2_O_2_ (IC_50_) (mg/mL)	FRAP (EC_50_) (mg/mL)	TAC (mg AAs/mL)
*M*. *piperita*	2.99 ± 0.20	0.96 ± 0.29	0.013 ± 0.003	0.101 ± 0.01	10.42 ± 0.03
*M*. *pulegium*	3.24 ± 0.20	1.02 ± 0.21	0.009 ± 0.008	0.42 ± 0.02	9.94 ± 0.21
*T. serpyllum*	1.39 ± 0.34	0.44 ± 0.7	0.05 ± 0.004	0.161 ± 0.002	7.25 ± 0.19
*T*. *zygis*	0.42 ± 0.07	0.40 ± 0.04	0.063 ± 0.01	0.09 ± 0.01	8.36 ± 0.42
BHT	0.021 ± 0.001	—	—	—	—
Gallic acid	—	0.019 ± 0.001	—	—	—
Ascorbic acid	—	—	0.023 ± 0.03	0.035 ± 0.010	—

## Data Availability

The data used in this study are included within the article.
